# Single-centre, prospective cohort to predict optimal individualised treatment response in multiple sclerosis (POINT-MS): a cohort profile

**DOI:** 10.1136/bmjopen-2025-103440

**Published:** 2025-09-25

**Authors:** Ronja Christensen, Alessandro Cruciani, Sarmad Al-Araji, Alessia Bianchi, Declan Chard, Samih Fourali, Weaam Hamed, Ahmed Hammam, Anna He, Baris Kanber, Davide Maccarrone, Marcello Moccia, Suraya Mohamud, Riccardo Nistri, Anestis Passalis, Valeria Pozzilli, Ferran Prados Carrasco, Eirini Samdanidou, Joy Song, Jed Wingrove, Charmaine Yam, Marios Yiannakas, Alan J Thompson, Ahmed Toosy, Yael Hacohen, Frederik Barkhof, Olga Ciccarelli, POINT-MS Group, Anuriti Aojula

**Affiliations:** 1 UCL Queen Square Institute of Neurology, UCL, London, UK; 2 Queen Square MS Centre, Department of Neuroinflammation, UCL, London, UK; 3 Department of Medicine and Surgery, Università Campus Bio-Medico di Roma, Rome, Italy; 4 Operative Research Unit of Neurology, Policlinico Universitario Campus Bio-Medico, Rome, Italy; 5 NIHR University College London Hospitals Biomedical Research Centre, London, UK; 6 Department of Human Neurosciences, University of Rome La Sapienza, Rome, Italy; 7 Department of Molecular Medicine and Medical Biotechnology, University of Naples Federico II, Naples, Italy; 8 Multiple Sclerosis Unit, Federico II University Hospital, Naples, Italy; 9 Department of Neurology, Great Ormond Street Hospital for Children, London, UK; 10 Hawkes Institute, Medical Physics and Biomedical Engineering Department, UCL, London, UK; 11 e-Health Centre, Universitat Oberta de Catalunya, Barcelona, Spain; 12 Cleveland Clinic London Ltd, London, UK; 13 Brain Repair and Rehabilitation, Institute of Neurology, UCL, London, UK; 14 Hawkes Centre, Faculty of Engineering, UCL, London, UK; 15 Radiology and Nuclear Medicine, Amsterdam University Medical Centres, Amsterdam, The Netherlands

**Keywords:** Multiple sclerosis, Clinical Decision-Making, Drug Utilization, Magnetic Resonance Imaging, Cognition

## Abstract

**Abstract:**

**Purpose:**

Multiple sclerosis (MS) is a chronic neurological condition that affects approximately 150 000 people in the UK and presents a significant healthcare burden, including the high costs of disease-modifying treatments (DMTs). DMTs have substantially reduced the risk of relapse and moderately reduced disability progression. Patients exhibit a wide range of responses to available DMTs. The Predicting Optimal INdividualised Treatment response in MS (POINT-MS) cohort was established to predict the individual treatment response by integrating comprehensive clinical phenotyping with imaging, serum and genetic biomarkers of disease activity and progression. Here, we present the baseline characteristics of the cohort and provide an overview of the study design, laying the groundwork for future analyses.

**Participants:**

POINT-MS is a prospective, observational research cohort and biobank of 781 adult participants with a diagnosis of MS who consented to study enrolment on initiation of a DMT at the Queen Square MS Centre (National Hospital of Neurology and Neurosurgery, University College London Hospital NHS Trust, London) between 01/07/2019 and 31/07/2024. All patients were invited for clinical assessments, including the expanded disability status scale (EDSS) score, brief international cognitive assessment for MS and various patient-reported outcome measures (PROMs). They additionally underwent MRI at 3T, optical coherence tomography and blood tests (for genotyping and serum biomarkers quantification), at baseline (i.e., within 3 months from commencing a DMT), and between 6–12 (re-baseline), 18–24, 30–36, 42–48 and 54–60 months after DMT initiation.

**Findings to date:**

748 participants provided baseline data. They were mostly female (68%) and White (75%) participants, with relapsing–remitting MS (94.3%), and with an average age of 40.8 (±10.9) years and a mean disease duration of 7.9 (±7.4) years since symptom onset. Despite low disability (median EDSS 2.0), cognitive impairment was observed in 40% of participants. Most patients (98.4%) had at least one comorbidity. At study entry, 59.2% were treatment naïve, and 83.2% initiated a high-efficacy DMT. Most patients (76.4%) were in either full- or part-time employment. PROMs indicated heterogeneous impairments in physical and mental health, with a greater psychological than physical impact and with low levels of fatigue. When baseline MRI scans were compared with previous scans (available in 668 (89%) patients; mean time since last scan 9±8 months), 26% and 8.5% of patients had at least one new brain or spinal cord lesion at study entry, respectively. Patients showed a median volume of brain lesions of 6.14 cm^3^, with significant variability among patients (CI 1.1 to 34.1). When brain tissue volumes z-scores were obtained using healthy subjects (N=113, (mean age 42.3 (± 11.8) years, 61.9% female)) from a local MRI database, patients showed a slight reduction in the volumes of the whole grey matter (−0.16 (−0.22 to –0.09)), driven by the deep grey matter (−0.47 (−0.55 to –0.40)), and of the whole white matter (−0.18 (−0.28 to –0.09)), but normal cortical grey matter volumes (0.10 (0.05 to 0.15)). The mean upper cervical spinal cord cross-sectional area (CSA), as measured from volumetric brain scans, was 62.3 (SD 7.5) mm^2^. When CSA z-scores were obtained from the same healthy subjects used for brain measures, patients showed a slight reduction in CSA (−0.15 (−0.24 to –0.10)).

**Future plans:**

Modelling with both standard statistics and machine learning approaches is currently planned to predict individualised treatment response by integrating all the demographic, socioeconomic, clinical data with imaging, genetic and serum biomarkers. The long-term output of this research is a stratification tool that will guide the selection of DMTs in clinical practice on the basis of the individual prognostic profile. We will complete long-term follow-up data in 4 years (January 2029). The biobank and MRI repository will be used for collaborative research on the mechanisms of disability in MS.

**STRENGTHS AND LIMITATIONS OF THIS STUDY**

- This is a large, prospective, real-world cohort of patients starting a disease-modifying treatment (DMT) in clinical practice, being followed up regularly over 5 years.
- All clinical, neuropsychological, imaging and patient-reported outcome data are being collected using standardised protocols consistent with standard-of-care in the NHS.
- Blood and serum samples were biobanked at each visit, enabling future biomarker and both genetic (i.e., at baseline) and epigenetic analyses.
- Single-centre recruitment may limit the generalisability of findings to other multiple sclerosis populations.
- Most participants started high-efficacy DMTs, reflecting current clinical practice in the UK, but limiting our ability to evaluate treatment response in lower efficacy drugs.

## Introduction

 Multiple sclerosis (MS) is a chronic neurological condition primarily of young adults,^1^ posing a substantial healthcare challenge, particularly in regions with high prevalence, such as the UK.^2^ MS incurs significant direct and indirect medical costs due to high costs of disease-modifying treatments (DMTs) and its progressive nature, often leading to long-term irreversible disability.^3^

The introduction of highly effective DMTs has improved the natural history of MS by dramatically reducing the risk of relapses and the presence of new lesions in follow-up MRI investigations. For example, ocrelizumab, which is a high-efficacy DMT, reduces the annualised relapse rate by approximately 46% and the number of new T2 lesions by 94%, when compared with interferon beta-1a in patients with relapsing–remitting MS (RRMS).^4^ The early use of high-efficacy therapies has been associated with improved outcomes in the long term.^5^,^7^ Although the impact of high-efficacy DMTs on the progression of disability has been moderate, continued treatment with immunotherapies reduces the risk of disability accrual in RRMS by 19%–44% over a period of 15 years.^8^

Patients show varied responses to DMTs, and complete disease control—defined as the absence of relapses, new lesions and disability progression—is not seen in all patients, even with high-efficacy treatments. A study in patients with RRMS treated with a high-efficacy DMT found that approximately 26% of patients did not achieve a complete disease control after 1 year^9^ and 34% after 3 years.^10^ Up to 12% of patients with RRMS develop disability progression independently of relapse activity (PIRA) every year.^11^ This suggests that acute inflammation is not the sole driver of disability accumulation and other mechanisms, which can be studied using imaging and serum biomarkers, can contribute to that.^11^ Individual patients’ characteristics, as defined by demographic variables, clinical, socioeconomic, lifestyle, imaging, genetic factors and serum biomarkers, may shape the individual response to a DMT and clinical outcomes.^12^,^15^

The primary aim of the Predicting Optimal INdividualised Treatment response in MS (POINT-MS) cohort is to develop a model that predicts the individualised treatment response to inform decision-making. This involves deeply phenotyping and genotyping a cohort of patients with MS initiating a DMT and using advanced statistical modelling and machine learning to predict the probability of responding to a DMT. Secondary aims are as follows: (1) improve our understanding of disease pathobiology by defining responders’ profile, which will enable a ‘reverse translation’ to more basic research; (2) understand the determinants of PIRA and (3) set up a biorepository to allow the discovery of new biomarkers of progression and further research studies on the mechanisms of disability. In this article, we describe the cohort’s profile at baseline and provide an overview of the study design.

## Cohort description

### Study design and eligibility criteria

**Figure 1:**
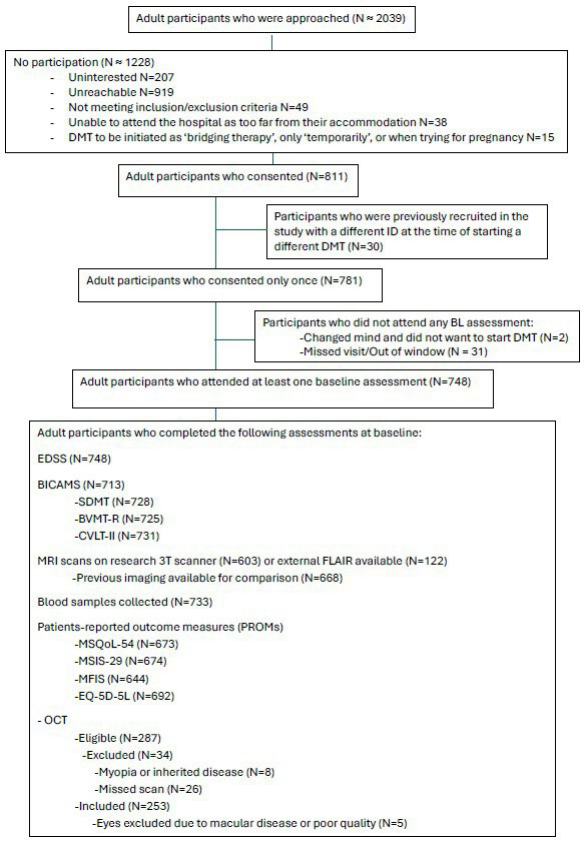
Participant flowchart. BICAMS, brief international cognitive assessment for MS; BL, baseline; BVMT-R, brief visuospatial memory test-revised; CVLT-II, California verbal learning test second edition; DMT, disease-modifying therapy; EDSS, expanded disability status scale; FLAIR, fluid-attenuated inversion recovery; MFIS, modified fatigue impact scale; MSIS-29, multiple sclerosis impact scale-29; MSQoL-54, multiple sclerosis quality of life scale-54; OCT, optical coherence tomography; SDMT, symbol digit modalities test.

This is a single-centre longitudinal observational cohort study including blood sample collection, brain and spinal cord MRI, clinical and neuropsychological evaluations and patient-reported outcomes. 2039 adult patients who were due to start a new DMT at the Queen Square MS Centre (University College London Hospital (UCLH) NHS Trust) between 01/07/2019 and 31/07/2024 were approached by the study team; 781 individuals met the inclusion criteria and consented to the study. Figure 1 details the participants’ flowchart.

The minimum required sample size was approximately 400 patients, determined by aiming for sensitivity and specificity estimates of~80% (±10%) for predicting NEDA. Calculations assumed NEDA rates of 45% for high-efficacy therapies (≥142 patients) and 25% for low-efficacy therapies (≥256 patients). Accounting for an expected dropout of approximately 5%–10% per year and to ensure representativeness, our desired target was ~700 patients. Recruitment ended pragmatically 6 months prior to funding completion.

The first study protocol, approved on 21/06/2019 (19/WA/0157), included three time points: a baseline visit (within 3 months from the initiation of a DMT), a rebaseline visit between 6 and 12 months and a final visit between 18 and 24 months. An optical coherence tomography (OCT) substudy was approved in 2021 via a substantial amendment (Sub_Amend_3) to invite participants to undergo OCT at baseline and then after 6–12 months and 18–24 months. While the original ethical approval in 2019 was to follow-up patients for 24 months after DMT initiation, in 2022, we obtained a new approval, through a non-substantial amendment (NSAmend6) to follow-up participants up to 54–60 months, if they reconsented to the study team accessing their clinical and imaging data collected during standard hospital visits. A subsequent ethics approval in 2023 (23/WS/0008) allowed further collection of participant data available from hospital electronic health records (independently of participants’ consent), which facilitated further data gathering, including filling in missing data points retrospectively.

Therefore, the final study design of the POINT-MS study included five time points: baseline, 6–12, 18–24, 30–36, 42–48 and 54–60 months after treatment initiation. At each visit, clinical assessments, blood samples and patient-reported outcome measures (PROMs) were collected. At their first three time points, patients were invited to undergo a brain and spinal cord MRI on the 3T research scanner at the Queen Square MS Centre and OCT. Clinical and MRI assessments at 30–36, 42–48 and 54–60 months are currently ongoing and integrated within the NHS clinical pathways as follows. (1) MRI scans are being acquired in the NHS with standardised sequences. (2) PROMs are electronically sent to patients 2 weeks prior to their hospital appointments. (3) The expanded disability status scale (EDSS) is scored by the treating neurologist during the appointments in the MS specialist clinics. (4) Blood samples are being collected on the day of the MRI and stored at −80 °C.

Inclusion criteria for the POINT-MS cohort were as follows: (1) a diagnosis of MS as per the 2017 McDonald criteria^16^ and (2) initiation of a new DMT within 3 months. Exclusion criteria were applied to specific tests; for cognitive assessment, the exclusion criteria were as follows: (1) use of steroid therapy in the last 3 months; (2) history of learning disability or major neurological or psychiatric condition and (3) use of cognitively altering drugs. For OCT, the exclusion criteria were as follows: (1) known ophthalmological diseases as per the OSCAR-IB criteria^17^; (2) refractive errors >6 or <−6 dioptres and (3) optic neuritis (ON) within the last 6 months.

Patients were identified through MS DMT initiation clinics and day-care unit. Participants were given an information sheet with the details of the POINT-MS study and separate invitations to participate in the main study and OCT substudy.

### Baseline visits

Baseline visits were carried out in-person. Clinical and demographic data, including disease onset, relapse history, DMT, lifestyle (smoking and illicit drug use) and socioeconomic (ie, income, occupational levels and years of education) data and comorbidities, were collected by patient interviews, review of medical records and use of study-specific digital tablets. Patients underwent physical examination, cognitive testing, MRI and had bloods drawn. OCT was only performed in those who consented to the OCT substudy. All clinical data and MRI reports were recorded using the REDCap platform hosted at the University College London (UCL) Queen Square Institute of Neurology, UCL.^18 19^ PROMs via questionnaires were collected during study visits^20^ or via email. Study visits were coordinated to occur on days when patients were, otherwise, coming to the hospital for clinic visits or infusion appointments to minimise participant burden. Where this was not possible, patients were reimbursed for travel expenses.

1. **Clinical assessments**

EDSS was performed by a trained neurologist.^21^ Cognitive performance was assessed using the Brief International Cognitive Assessment for MS (BICAMS)^22^ comprising the written Symbol Digit Modalities Test (SDMT) as a measure of processing speed, the first three recall trials of the Brief Visuospatial Memory Test-Revised (BVMT-R) as a measure of visuospatial learning and memory and the first five recall trials of the California Verbal Learning Test Second Edition (CVLT-II) as a measure of verbal learning and memory. Healthy controls were recruited as a part of a larger longitudinal study on cognitive function in MS, approved by the UCL Research Ethics Committee (Ethics number: 28405/002), and written consent was obtained from all participants. Age, sex and years of education were used to generate demographically adjusted z-scores for each patient based on our own healthy control sample (N=53; mean age 30 years (SD 13), years of education 15.21 (SD 2.44) and 57% female (see online supplemental material S1 for procedure and online supplemental table S1) for descriptive statistics). A test score was classified as impaired if it fell below≥1.5 SD from the mean. All patients who consented to the OCT substudy were also assessed for best-corrected visual acuity (VA). High-contrast VA was assessed monocularly with and without pinhole correction using the logarithm of the minimum angle of resolution acuity chart. Low-contrast VA was assessed using a lightbox LCVA chart at 2.5% and 1.25% contrast levels.

1. **MRI scans**

All patients were invited to undergo an MRI scan on the MS Research scanner, a 3-Tesla Philips Ingenia CX 3T MRI System (Philips Medical Systems, Best, Netherlands), equipped with a neurovascular 16-channel coil for brain imaging, integrated with total spine coils. Acquisition parameters for the brain and spinal cord MRI are shown in online supplemental table S2. All scans were uploaded to an open-source imaging informatics platform, XNAT (https://www.xnat.org/), under a pseudoanonymised study ID. All POINT-MS MRI scans were donated to UCLH to support patient care and were reported by the study radiology team. For patients who were not able to attend the research unit for their study MRIs, NHS hospital MRIs acquired with a standardised protocol were retrieved from the hospital PACS system and transferred to XNAT for analysis. The images were preprocessed as follows. (1) Lesions were identified using the automated lesion delineation software, nicMS,^23^ with 3-D T2-fluid-attenuated inversion recovery (FLAIR) images as input; all lesion masks were manually reviewed and adjusted as needed by trained analysts under the supervision of a senior neuroradiologist (FB), and lesion load was calculated as the total volume of focal lesions. (2) Next, lesion filling was applied to 3D T1-turbo field echo images. (3) Then, the images were segmented into grey matter (GM) and white matter (WM) using the Geodesic Information Flow software,^24^ and tissue volumes were extracted using NiftySeg software. All tissue volumes were adjusted by total intracranial volume. (4) Brain volumes, already normalised for intracranial volume, and upper cervical spinal cord cross-sectional area (CSA) were standardised using a linear regression model based on age and sex, derived from a dataset of 113 healthy controls (mean age 42.3 (SD 11.8) and 61.9% female) (online supplemental table S3) whose scans were available in the MRI database of the Queen Square MS Centre, Department of Neuroinflammation, UCL. Z-scores were computed by subtracting the model-predicted value from the observed value and dividing by the SD of the residuals in healthy controls. (5) For the computation of upper cervical spinal cord CSA, 3D-T1 images of the brain were used;^25^ in brief, the top of C2 vertebral body and the bottom of C3 vertebral body were first manually marked by trained analysts and the CSA was subsequently segmented automatically using the DeepSeg algorithm available with the Spinal Cord Toolbox.^25^

### Blood samples collection

Blood was drawn by one of the doctors and/or nurses who were trained in phlebotomy. Two 6 mL EDTA tubes and one 5 mL serum tube (SST II advance) of blood were drawn from each patient at baseline. The serum tube and one EDTA tube were centrifuged using the Eppendorf Centrifuge 5810R at 3000 x g at room temperature for 5 min. Following this, 500 µL were pipetted into each of 6×2 mL PCR-PT Sarstedt aliquots. The second EDTA tube was frozen unprocessed. Samples were processed and frozen in an onsite freezer at −80° for future analyses.

1. Questionnaires

PROMs were collected using five validated questionnaires. (1) The MS quality of life (MSQOL-54) comprising 54 items related to subjective health-related quality of life.^26^ (2) The MS impact scale (MSIS-29) comprising 29 questions regarding the physical and psychological impact of MS^27^. (3) The EQ-5D-5L measuring mobility, self-care, usual activities, pain/discomfort and anxiety/depression^28^. (4) The modified fatigue impact scale (MFIS) assessing the impact of fatigue on physical, cognitive and psychosocial functioning^29^. (5) The work productivity and activity Impairment assessing health-related absenteeism, presenteeism and impairments in unpaid activities.^30^ In addition, all female participants were asked targeted women’s health questions cross sectionally, including age of menarche/menopause, pregnancy history, miscarriage history, Hormone Replacement Therapy (HRT) and contraceptive use.

1. Optical Coherence Tomography

OCT was performed on a Heidelberg Spectralis OCT2 machine with non-invasive angiography capabilities and Spectralis software V.7.0.1. The instrument used 1024 A-scan points with a 3.45 mm circle centred on the optic disc. The acquisition rate was 40 000 A-scans per second at an axial resolution of 3.9 μm. A peripapillary retinal nerve fibre layer scan was performed using the axial protocol centred around the optic nerve head, with ART set to 100. A volumetric (20° × 20° volume) scan of the macula centred on the fovea was then performed (73 B-scans covering a superior–inferior distance of 4.6 mm). Macular thicknesses were obtained from the 6 mm ring of a 1, 3 and 6 mm early treatment diabetic retinopathy study circular *grid* map.

OCT scans were quality checked using the OSCAR-IB criteria before extraction.^17^ Scans that did not meet the criteria, or had a signal strength of<25, were excluded from the analysis. Eyes affected by a recent episode of ON (within 6 months of the OCT scan, severe refractive error (> + or <− 6 dioptres)) or had significant retinal pathology as per the OSCAR-1B criteria, which may affect interpretation of the OCT, were excluded.

### Patient and public involvement (PPI)

PPI has been integral to the design and implementation of this study, ensuring that the research aligns with the priorities and experiences of individuals living with MS. A small PPI group has contributed to draft the patient information sheet. Regular PPI (virtual) meetings have been held throughout the study period to discuss the progress and the preliminary results of our research.

## Findings to date

We present some descriptive results of the baseline data for the purpose of introducing the cohort and illustrated the data available to the research community.

### Characteristics of study participants

Demographic and clinical characteristics of the patients at study entry are reported in table 1. The cohort consisted of 748 participants (mean age 40.8 (SD 10.9) years), most of whom were females (68%), White (75.1%) and with RRMS (94.3%). Patients had a relatively short disease duration (mean 7.9, SD 7.4, years since symptom onset). Of the total sample, 38.64% (N=289) were ex-smokers, while 10.96% (N=82) were current smokers. Most patients (98.4%) had at least one comorbidity. Median EDSS at baseline was 2 (IQR 2), indicating generally mild but variable levels of disability.

**Table 1:** Baseline clinical and demographic statistics

	N=748
Age, years, mean (SD)	40.8 (10.9)
Sex, N (%) females	509 (68%)
Ethnic/racial groups	
White	561 (75%)
Black	46 (6.1%)
Asian	60 (8%)
Mixed	40 (5.3%)
Other	41 (5.5%)
Phenotypes	
RRMS	705 (94.3%)
SPMS	7 (0.9%)
PPMS	36 (4.8%)
Disease duration, months (SD)*	95.2 (88.5)
Years of education, mean (SD)	15.3 (2.5)
Lifestyle	
Smoking, n (%)	
Current	82 (10.96%)
Ex-smoker	289 (38.64%)
Illicit drug use, n (%)	52 (6.9%)
No of comorbidities	
0	12 (1.6%)
1	544 (72.7%)
2	177 (23.7%)
3	13 (1.7%)
4	2 (0.3%)
Clinical relapses in the past 2 years, mean (SD)	1.2 (1.1)
EDSS, median (range)	2 (0–8)
BICAMS, mean (SD)(% impaired)	
SDMT	51.6 (13.9)(28%)
BVMT-R	24.4 (7.5)(24%)
CVLT-II	51.9 (11.5)(17.6%)
Treatment-naïve patients	443 (59.2%)
Number of patients with previous DMTs (%)	
1	171 (22.9%)
2	83 (11.1%)
3	35 (4.7%)
4	11 (1.5%)
5	5 (0.7%)
DMT history, mean (SD)	
Proportion of DD on DMT	0.23 (0.32)
Proportion of DD on HE-DMT	0.04 (0.12)
Proportion of DD on NHE-DMT	0.19 (0.30)
New DMT	
Non-high efficacy	126 (16.8%)
Glatiramer acetate	30 (4%)
Interferon	11 (1.5%)
Dimethyl fumarate	24 (3.2%)
Fingolimod	5 (0.7%)
Siponimod	5 (0.7%)
Teriflunomide	2 (0.3%)
Cladribine	49 (6.5%)
High efficacy	622 (83.2%)
Ocrelizumab	386 (51.5%)
Ofatumumab	226 (30.1%)
Natalizumab	9 (1.2%)
Alemtuzumab	1 (0.1%)
Women’s health information† (N=256)	
Age at menarche, mean (SD)	12.86 (3.49)
Number of women ever pregnant, N (%)	134 (52.34%)
Number of live births, mean (SD)	1.46 (1.05)
Menopause, N (%)	38 (14.84%)
Age at menopause, mean (SD)	48.07 (9.31)

* Since symptom onset.

† Collected using a patient-reported questionnaire.

BICAMS, brief international cognitive assessment for MS; BVMT-R, brief visuospatial memory test-revised; CVLT-II, California verbal learning test second edition; DD, disease duration; DMT, disease-modifying therapy; EDSS, expanded disability status scale; PPMS, primary progressive MS; RRMS, relapsing–remitting MS; SDMT, symbol digit modalities test; SPMS, secondary progressive MS.

**Figure 2:**
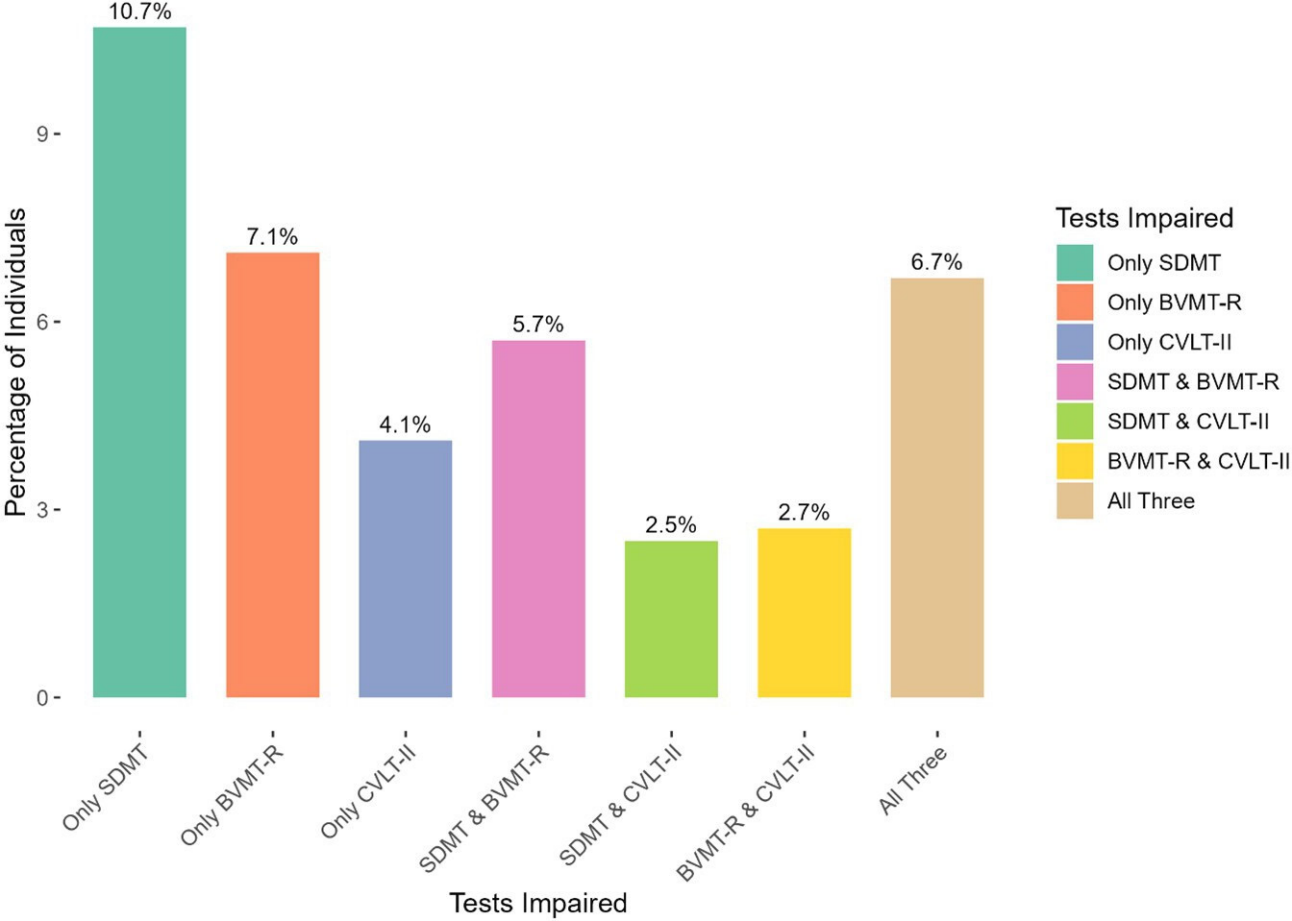
Impairment combinations for BICAMS subtests. BICAMS, brief international cognitive assessment for MS; BVMT-R, brief visuospatial memory test-revised; CVLT-II, California verbal learning test-second edition; SDMT, symbol digit modalities test.

On BICAMS test of cognitive performance, 301 (40.2%) of the participants showed impairment in at least one test (table 1); 28% were classified as impaired on the SDMT, 24% on the BVMT-R, while 17.6% scored below the cut-off on the CVLT-II. When comparing impairments between the BICAMS subtests, 10.7% of participants were impaired only on the SDMT, 7.1% on BVMT-R and 4.1% (N=31) on CVLT-II, while 5.8% were impaired on both SDMT and BVMT-R, 2.5% (N=19) on both SDMT and CVLT-II, while 2.7% (N=20) impaired on both BVMT-R and CVLT-II (figure 2).

**Figure 3:**
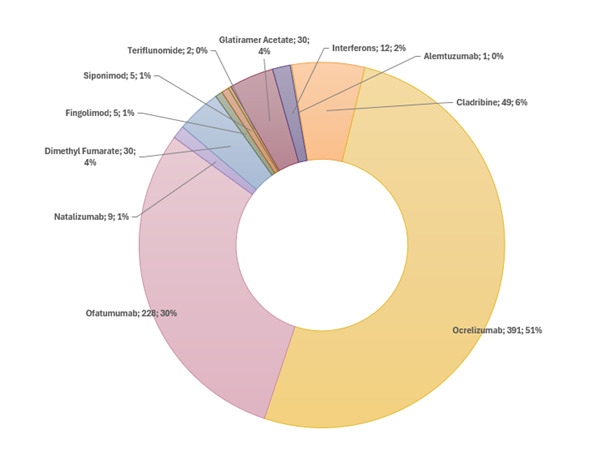
Distribution of initiated disease-modifying therapies in the POINT-MS cohort. POINT-MS, Predicting Optimal INdividualised Treatment response in MS.

At study entry, 59.2% of participants were treatment naïve, and 83.2% initiated a high-efficacy DMT. The drug, which was most frequently initiated, was ocrelizumab (51.4%), followed by ofatumumab (30.1%) (figure 3). At baseline, participants had spent an average of 22.7% (SD 31.6%) of their disease duration (calculated from symptom onset until day of baseline visit) on any DMT. The average proportion of time spent on high-efficacy DMTs was 3.9% (SD 12.1%), while 18.8% (SD 30.3%) of disease duration was spent on non-high-efficacy DMTs (table 1).

Employment and education are reported in table 2. Of the 725 patients who provided information about their current employment during the baseline interview, 76.4% were employed, with mean 35.1 (SD 12.8) hours worked per week. For the remaining 23.6% who reported no employment, 66 (38.4%) defined themselves as unemployed, 22 (12.8%) were permanently sick/disabled, 18 (10.5%) were temporarily sick/disabled, 28 had retired (16.3%), 13 (7.6%) were in formal education, 20 (11.6%) were looking after home/family, 4 (2.3%) were on maternity leave and 1 reported voluntary redundancy.

**Table 2:** Employment and occupations (N=725)

Income, £/month before deductions, mean (**SD**)		**3161.3 (1487.3)**
**Occupational status, employed, N%**		554 (76.4%)
**Hours worked per week, mean (SD**)		35.1 (12.8)
Current occupational levels*		
	**Administrative and secretarial occupations**	60 (10.8%)
	**Associate professional and technical occupations**	50 (9%)
	**Caring, leisure and other service occupations**	20 (3.6%)
	**Elementary occupations**	7 (1.3%)
	**Managers, directors and senior officials**	171 (30.9%)
	**Process plant and machine operatives**	1 (0.2%)
	**Professional occupations**	176 (31.8%)
	**Sales and customer service occupations**	29 (5.2%)
	**Skilled/trade occupations**	26 (4.7%)
	**Not applicable**	14 (2.5%)

* Patients currently employed.

PROMs are reported in table 3. The group of 673 patients who completed the MSQoL-54 questionnaires reported moderate levels of physical health, emotional well-being and mental health, although with large variance.^26^ Similarly, scores on MFIS fatigue (MFIS total) indicate a level of fatigue comparable with other studies in RRMS,^31 32^ however again with large variation. On the MSIS, the psychological impact of MS was greater than the physical impact, indicating a stronger emotional burden. In the overall EQ-5D-5L index, participants had (on average) a favourable health perception, although a substantial number experienced significant disease-related impairments.

**Table 3:** PROMs at baseline

MSQOL-54, mean (SD)	N=673
Physical health	73.31 (29.39)
Role limitations due to physical problems	55.33 (43.98)
Role limitations due to emotional problems	65.07 (41.68)
Pain	74.09 (25.29)
Emotional well-being	63.41 (20.42)
Energy	43.58 (21.18)
Health perceptions	51.25 (20.99)
Social function	70.17 (24.51)
Cognitive function	66.39 (26.22)
Health distress	50.13 (27.45)
Sexual function	57.19 (21.69)
Change in health	42.85 (23.09)
Satisfaction with sexual function	57.06 (31.68)
Overall quality of life	68.00 (18.41)
Physical health composite	63.48 (20.33)
Mental health composite	64.52 (22.58)
MSIS-29, mean (SD)	N=674
Physical impact	24.71 (23.69)
Psychological impact	34.91 (23.65)
Total	27.69 (21.82)
MFIS, mean (SD)	N=664
Physical	16.86 (10.80)
Cognitive	15.31 (10.33)
Psychosocial	3.02 (2.45)
Total	33.27 (20.83)
EQ-5D-5L values, mean (SD)	N=692
Mobility	0.04 (0.06)
Self-care	0.02 (0.03)
Activities	0.04 (0.05)
Pain	0.06 (0.07)
Anxiety/depression	0.08 (0.08)
EQ index (0 death to 1 perfect health)	0.76 (0.21)
EQ-VAS	72.07 (19.13)
WPAI, mean (SD)	N=624
Working, yes N(%)	480 (74.21%)
Percent work missed	9.72 (24.91)
Percent impairment while working	51.26 (35.77)
Percent overall work impairment	52.08 (35.42)
Percent activity impairment	46.49 (33.28)

MFIS, modified fatigue impact scale; MSIS-29, multiple sclerosis impact scale-29; MSQOL-54, multiple sclerosis quality of life scale-54; PROMs, patient-reported outcome measures; WPAI, work productivity and activity impairment.

MRI characteristics are summarised in table 4. Most patients (N=603, 80.6%) underwent MRI scans on the 3T research scanner. Of the remaining patients, 122 (16.3%) were scanned on a 3T NHS scanner, while 23 (3.1%) did not have FLAIR scan available. Previous imaging was available for comparison in 668 patients (89.3%). At baseline, 26% of patients had new brain lesions and 8.5% had new spinal cord lesions compared with their previous most recent clinical scans. The mean interval since the prior scan was 9 months (SD 8.2, range: 0–80 months). Among patients with new brain lesions, the mean interval was 10.4 months (SD 9.4), compared with 9 months (SD 7.4) for those without new lesions. Similarly, patients with new spinal cord lesions had a mean scan interval of 10.8 months (SD 9.9) versus 9 months (SD 8.0) for those without. The majority (642 patients; 86%) showed at least one cervical spinal cord lesion at baseline. We obtained volumetric brain measures using only the 3D-T1 research scans (N=599, since in 4 subjects, the analysis failed because of movement artefacts); none of the NHS scans include 3D-T1 (online supplemental figure 1). The volumetric measures were standardised using a reference healthy population composed of 113 healthy controls (mean age 42.3 (SD 11.8) and 61.9% female) (online supplemental table 3). The resulting z-scores indicated that the median z-score of the whole WM volume was −0.18 (95% CI (−0.28 to −0.11)), which was similar to that of the whole GM volume (−0.16 (95% CI (−0.22 to −0.09)) indicating that both the whole WM and GM volumes were almost at the mean of those of the reference healthy population. The deep grey matter (DGM) volume was smaller than the reference healthy population (median z-score: −0.47, 95% CI (−0.55 to –0.40)). Surprisingly, the cortical grey matter volume was normal (median z-score: 0.10, 95% CI (0.05 to 0.15)). The total lesion volume and the total number of lesions were calculated on the baseline 3-D FLAIR sequences (considering all together research and NHS scans) in 715 patients (online supplemental figure 1); the median total lesion volume was 6.14 cm^3^ (95% CI (1.17 to 34.10)), and the median number of lesions was 42 (95% CI (10 to 196)), with considerable variability among patients (table 4). Cervical spinal cord showed a CSA of 62.3 (7.5), which, when z-scored according to the same healthy controls used for brain volumetrics, showed a slightly reduced CSA (median z-score: −0.15 (−0.24 to –0.10)).

**Table 4:** MRI baseline characteristics

		N=748
Time since previous scan, months, mean (SD) (N=668)		9.01 (8.17)
Number of new brain lesions, number (%) (N=748) Number of brain enlarging lesions, number (%) (N=748) Patients with at least one cervical spinal cord lesion, number (%) (N=748) Number of new spinal cord lesions, number (%) (N=748)		196 (26%) 21 (2.8%) 642 (86%) 64 (8.5%)
3D-T1 cervical CSA, mean (SD), SD as percentage of cervical CSA (N=597) 3D-T1 cervical CSA, z-score, median (CI) (N=597)		62.3 (7.5) (12%) −0.15 (−0.24 to −0.10)
FLAIR brain measures (N=715)		
	Total lesion volume, cm^3^, median (CI)	6.14 (1.1 to 34.1)
	Total number of lesions, number, median (CI)	42 (10 to 196)
3D-T1 brain measures, z-scores, median (CI) (N=599)		
	WMn	−0.18 (−0.28 to −0.11)
	GMn	−0.16 (−0.22 to −0.09)
	CGMn DGMn	0.10 (0.05 to 0.15) −0.47 (−0.55 to −0.40)

CGMn, normalised cortical grey matter; CSA, cross-sectional area; DGMn, normalised deep grey matter; FLAIR, fluid-attenuated inversion recovery; GMn, normalised grey matter; WMn, normalised white matter.

The OCT data are summarised in table 5. Of 287 eligible patients, a total of 253 patients underwent OCT at study entry. They contributed 501 eyes that passed criteria. There were 114 ON eyes based on clinical history.

**Table 5:** Summary statistics for OCT measures

OCT layer (microns)	All MS eyes (N=501)	ON eyes (N=114 eyes)	NON eyes (N=387 eyes)
GCIPL, mean (range)	74.12 (66.88–79.50)	63.88 (55.78–71)	75.88 (70.88–80.81)
pRNFL, mean (range)	92 (83–102)	80 (69–91)	95 (87–104)
INL, mean (range)	37.25 (35.50–39.12)	37.81 (35.41–39.78)	37.25 (35.50–38.88)

GCIPL, ganglion cell-inner plexiform layer; INL, inner nuclear layer; MS, multiple sclerosis; NON, non-optic neuritis; OCT, optical coherence tomography; ON, optic neuritis; pRNFL, peripapillary retinal nerve fibre layer.

### June 2025 update on the POINT-MS cohort

Of these, 704 (94%) have been seen at 6–12 months, 544 (73%) at 18–24 months and 84 (11.2%) at 54–60 months (the latter two time points are still ongoing). Reasons for missed visits are being recorded and reviewed by the study team. As previously noted, ethical approval (REC 23/WS/0008) allows retrospective access to electronic health records, enabling us to fill in data gaps (eg, EDSS scores from clinical appointments and NHS-acquired MRIs) and extract additional clinical outcomes (eg, serious infections, hospital admissions or cause of death) for participants who remain under the care of our NHS Trust but do not attend study visits. For participants truly lost to follow-up—such as those who move abroad or transfer to a different NHS Trust—appropriate methods for handling missing data will be selected based on the study’s analytical objectives, and sensitivity analyses will be conducted accordingly.

## Discussion

The baseline characteristics of the POINT-MS cohort revealed interesting observations and align closely with those of some single-centre or multicentre (national) cohorts and anti-CD20 trials in RRMS. In particular, patients’ age (mean 48.8 years (SD 10.9) in our cohort) was similar to that of the EPIC (UCSF, USA) cohort (mean 41.6 years (SD 9.7),^33^ FutureMS (Scotland) cohort (mean age at diagnosis 37.7 years)^34^ and anti-CD20 trials (37.1 years in the ocrelizumab trial^4^ and 38.9 years in the ofatumumab trial).^35^ The percentage of female patients was also similar (68% in our cohort, 70.4% in the EPIC cohort and 75.4% in the FutureMS cohort). POINT-MS patients’ disability was mild (median EDSS=2), which was the same as that of patients in the FutureMS cohort, and similar to that of patients enrolled in the EPIC cohort (median EDSS 1.5), ocrelizumab (mean 2.86) and ofatumumab (mean 2.97) trial, although the EDSS range in the POINT-MS cohort was wider (up to EDSS 8).

The POINT-MS cohort showed a 40% rate of cognitive impairment, defined as failure on a single test. This agrees with previous reports^36 37^ and underscores the substantial cognitive burden even among patients with relatively mild physical disability (as assessed by the EDSS). We found the biggest impairment on the SDMT, followed by BVMT-R, while the CVLT-II was relatively unimpacted. The finding that such impairment was detected in subtests other than the SDMT confirms the importance of administering the entire BICAMS battery to capture key components of cognitive dysfunction in MS, with a minimum of SDMT-BVMT-R, as previously suggested.^38^ Despite cognitive impairment, the percentage of patients in employment is 76.4%, which is on par with the employment rate for people aged 16–64 in the UK (2024 data from the Office for National Statistics). This rate of employment is slightly higher than previous reports, where employment ranges from 54% at EDSS 3 to 77% at EDSS 1.^39^ One possible explanation for this result may lie in the relatively short disease duration (mean 7.9 years) and the large proportion of patients (83.2%) who initiated MS treatment with high-efficacy DMTs, in line with National Guidelines,^40^ compared with just 16.8% on moderate and low-efficacy treatments. Several studies have demonstrated that starting with high-efficacy DMTs can reduce disability accrual over time, resulting in significantly better outcomes for patients and significant reductions in healthcare costs.^41^ Combining the current and ex-smokers of our cohort comes to 49.6%, highlighting a notably greater historical exposure to smoking compared with the UK general population (24.9%).^42^ This is a concerning trend, given the well-documented impact of smoking on the overall health, and its association with disease progression.^43^ Encouragingly, only 10.96% were still smokers at the time of data collection, which is a positive finding, as smoking cessation may slow disease progression to a rate comparable with that of never smokers.^44^

The racial/ethnic diversity of the cohort reflects the clinical catchment of central London. Of the participants, 189 (24.9%) identified themselves as belonging to a non-White ethnic group, with Asian and Black individuals being the most represented among them.

Most of the POINT-MS patients were treatment naïve (59.2%) and initiated a high-efficacy DMT (83.2%), despite low to moderate physical disability, indicative of a growing tendency for clinicians to prescribe high-efficacy medications.^5^ Nonetheless, over 40% of patients were enrolled when switching from another DMT, and nearly, 20% had yet to find a suitable treatment after trying two or more DMTs, highlighting the critical need for predictive tools based on deeply phenotyped patients to guide individualised treatment decisions and improve long-term outcomes.

MRI characteristics also offer numerous interesting insights. At baseline, 26% of participants showed new T2 lesions in the brain and 8.5% in the spinal cord, compared with previous scans, mostly performed in the NHS, which indicates a moderately high level of radiological activity, as expected to be seen in patients initiating a new DMT, although smaller than the proportion of patients showing gadolinium enhancing lesions in the ocrelizumab (42.5%)^4^ and ofatumumab (37.8%)^35^ trials. The brain lesion load of POINT-MS patients was slightly lower than those of patients recruited in the anti-CD20 clinical trials (6.14 cm^3^ vs 10 cm^3^ and 13 cm^3^), with a high variability among patients,^4 35^ although comparisons should be taken with caution as different MRI acquisition and analysis methods were used.

The analysis of brain lesions and tissue volumes revealed that the POINT-MS cohort showed a very mild atrophy in the whole brain WM and a mild atrophy in the DGM, which has been reported before in patients with RRMS.^45^

Interestingly, the percentage of patients with cervical spinal cord lesions was 86%, which is slightly higher than previously seen in RRMS cohorts,^46^ despite a relatively low level of disability (median EDSS 2.0). In addition, we found that spinal cord CSA was relatively homogeneous across the studied population, with an SD of 7.5 (approximately 12% of the mean cervical CSA of 62.33 mm^2^). This suggests low intersubject variability, particularly when compared with the wide 95% CIs for lesion load (1.1 to 34.1 cm³) and lesion number (10 to 196) in our population. While this CSA appears lower than those reported in previous similar studies,^47^ comparisons should be made cautiously due to differences in cervical cord levels and analysis pipelines. Additionally, the CSA z-score analysis suggested a minimal degree of spinal cord atrophy across the cohort.

This study has several methodological strengths. The prospective, standardised design across a large sample enables robust, longitudinal analyses of treatment outcomes and disease progression. Multimodal data were collected in a real-world clinical setting by a trained team using consistent and standardised protocols. Blood and serum samples were biobanked at each visit, enabling future biomarker and genetic studies. Additionally, patients were involved in the design of this cohort. However, our study is not without limitations. First, recruitment from a single specialist centre may limit generalisability to broader MS populations or healthcare systems, although the single-centre designs allowed selection of a more homogeneous (highly selected) population than in multicentre studies, which is expected to contribute to a larger intervention effect than that observed in multicentre studies. Treatment choice was discussed in multidisciplinary meetings, and this mitigated the risk of idiosyncrasy in the prescription of DMTs. Second, the predominance of patients starting high-efficacy DMTs constrains our ability to evaluate treatment response in low- and moderate-efficacy drugs.

While many MS data cohorts exist globally, including large-scale (inter)national registries (eg, MSBase, the Danish, Italian and Swedish MS Registries), these are typically retrospective and often rely on opportunistic and variable clinical data. In contrast, POINT-MS includes a deeply phenotyped, prospective cohort with harmonised clinical, imaging and blood/serum collection protocols across follow-up. This design aligns more closely with other prospective single-centre research cohorts, such as the EPIC, CLIMB and FutureMS cohorts. Compared with these, POINT-MS is unique in its systematic multimodal integration, real-world treatment setting and prevalence of high-efficacy DMT initiators. Importantly, POINT-MS provides complementary data to clinical trial populations, particularly those in anti-CD20 studies, while still reflecting real-world diversity and treatment patterns.

In conclusion, the study design, large sample size and high-quality data collected within the POINT-MS cohort provide a valuable resource for gaining deeper insights into the characteristics and disease trajectories of patients with MS. In this evolving landscape, characterised by a diverse range of drugs with distinct mechanisms of action, future research will play a pivotal role in personalising treatment and identifying the optimal therapy for each patient.

## Collaboration

We welcome potential collaborators to engage with the POINT-MS study or related research on individualised treatment response and mechanisms of disability in MS. This includes opportunities for data sharing, pooled or harmonised analyses and hypothesis-driven substudies, as well as collaborative contributions to imaging, clinical and biomarker research. Anonymised and tabulated data from the POINT-MS cohort can be requested by contacting the principal investigator (OC).

## Supplementary material

10.1136/bmjopen-2025-103440 online supplemental file 1

## Data Availability

Data are available on reasonable request.
