# Supplementary material for: Single-centre, prospective cohort to predict optimal individualised treatment response in multiple sclerosis (POINT-MS): a cohort profile
Source: BMJ Open. 2025 Sep 25;15(9):e103440. doi: 10.1136/bmjopen-2025-103440 (PMC12481372; doi:10.1136/bmjopen-2025-103440)
Supplement: online supplemental file 1 [file bmjopen-15-9-s001.docx]

**Supplementary Materials**

**Supplementary Materials S1**

**S1: Recruitment, protocol and z-score calculations for healthy controls**

**Recruitment and procedure**

We used data from healthy controls (N = 53) recruited as part of a larger study investigating longitudinal cognitive performance in healthy controls. The only inclusion criterion required participants to be aged between 18-65, while exclusion criteria were: (1) history of neurological or psychiatric diseases (such as depression or anxiety), (2) use of cognitively altering medication (such as antidepressants or lisdexamfetamine, or (3) use of illicit drugs which may alter cognitive state (such as cannabis or cocaine). This independent study was approved by the UCL Research Ethics Committee on the 21^st^ of August 2024 (ethics number: 28405/002). All participants provided informed written consent.

All participants were invited for a study visit at baseline and underwent testing with the Brief International Cognitive Assessment (BICAMS)^1^, comprising the Symbol Digit Modalities Test (SDMT), the Brief Visuospatial Memory Test-Revised (BVMT-R) and the English version of the California Verbal Learning Test-Second Edition (CVLT-II). The Mini-Addenbrooke’s Cognitive Examination (M-ACE)^2^ was used as a screening measure for dementia. Demographic information (age, sex, years of education) was also collected.

**Z-score calculation and definition of impairment**

All participants scored above 25 on the M-ACE and were included in the analysis. There were no significant differences between patients and controls in age (t (56.7) =1.36, *p* = 0.18)), years of education (t(60.5) = 0.32, *p* = 0.75)) or sex distribution (X^2^ = 2.45, *p* = 0.12).

We calculated demographically adjusted z-scores for each BICAMS subtest (SDMT, BVMT-R, and CVLT-II) using our internal sample of healthy controls (N = 53). Multiple linear regression models were built separately for each test score using age, sex, and years of education as predictors. Predicted scores were generated for each patient based on their demographics, and residuals were computed as the difference between observed and predicted values. These residuals were then standardised using the SD of the residuals from the control group, yielding z-scores that reflect performance relative to demographically expected norms.


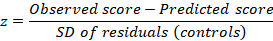


Cognitive impairment was defined as a z-score less than -1.5 on any given test (i.e., ≥1.5 SD below the demographically adjusted mean. This threshold is commonly used in neuropsychological research and reflects clinically meaningful deviation from expected cognitive performance. Group-level impairment was further categorised as “Impaired” or “Preserved” for each subtest based on this cut-off.

**Supplementary Table 1: Descriptive statistics in patients and controls**

|  | Patients (N = 748) | Controls (N = 53) |  |
| --- | --- | --- | --- |
| Age, mean (SD) | 40.78 (10.86) | 38.16 (13.70) | 0.18 |
| Sex, n% female | 509 (68.05%) | 30 (56.6%) | 0.12 |
| Years of education | 15.32 (2.55) | 15.21 (2.44) | 0.75 |
| SDMT | 51.62 (13.95) | 59.34 (10.51) |  |
| BVMT-R | 24.39 (7.52) | 28 (5.87) |  |
| CVLT-II | 52.86 (11.53) | 54.57 (9.10) |  |

**References**

1. Langdon DW, Amato MP, Boringa J, et al. Recommendations for a brief international cognitive assessment for multiple sclerosis (BICAMS). *Multiple Sclerosis Journal*. 2012;18(6):891-898. doi:10.1177/1352458511431076

2. Hsieh S, McGrory S, Leslie F, et al. The Mini-Addenbrooke’s Cognitive Examination: A New Assessment Tool for Dementia. *Dement Geriatr Cogn Disord*. 2015;39(1-2):1-11. doi:10.1159/000366040

**Supplementary Table 2.** The acquisition parameters used for brain and spinal cord imaging used on the 3T Philips scanner (N=599 patients).

|  | **Imaging protocol** | | | | |
| --- | --- | --- | --- | --- | --- |
|  | ***Brain imaging*** | | ***Spinal cord imaging*** | | |
| **Pulse sequence** | **3D T2-weighted FLAIR** | **3D T1-weighted TFE** | **2D PD-weighted TSE** | **2D T2-weighted STIR** | **2D T2-weighted TSE** |
| **RF coil** | SENSE NV 16 channel | SENSE NV 16 channel | SENSE Spine 15 channel | SENSE Spine 15 channel | SENSE Spine 15 channel |
| **Plane** | Sagittal | Sagittal | Sagittal | Sagittal | Axial |
| **TR [ms]** | 4800 | 7 | 2500 | 4000 | 4000 |
| **TE [ms]** | 267 | 3.2 | 29 | 80 | 120 |
| **Flip angle/refocussing [degrees]** | 180/40 | 8 | 90 | 180/120 | 90/120 |
| **Tl [ms]** | 1650 | 823 | N/A | 250 | - |
| **NEX** | 2 | 1 | 1 | 1 | 1 |
| **TSE/TFE factor** | 182 | 225 | 8 | 24 | 29 |
| **Slice number** | 150 | 176 | 15 | 15 | 15 |
| **Slice thickness [mm]** | 1.2 | 1 | 3 | 3 | 3 |
| **FOV [mm^2^]** | 256×256 | 256×256 | 260 × 479 | 260x479 | 150x150 |
| **Voxel size [mm^3^]** | 1.2x1.2x1.2 | 1x1x1 | 0.9x0.9x3 | 0.9x0.9x3 | 0.75x1x3 |
| **SENSE factor (phase/**  **slice)** | 3/2 | - | 2.5 | 2 | - |
| **Compressed SENSE factor** | - | 6 | - | - | - |
| **Scanning time [min]** | 3:36 | 1:56 | 4:30 | 3:04 | 4:16 |

FLAIR: Fluid-Attenuated Inversion Recovery; TFE: Turbo Field-Echo; TSE: Turbo Spin-Echo; STIR: Short Tau Inversion Recovery; PD: Proton Density; TR: Repetition Time; TE: Echo-Time; TI: Inversion Time; NEX: Number of Excitations; FOV: Field of View; SENSE: Sensitivity Encoding; RF: Radio-frequency.

**Supplementary Table 3.** Clinical and MRI characteristic of the healthy control population

|  | |  |  | N = 113 |
| --- | --- | --- | --- | --- |
| Age, mean (SD) | | | | 42.3 (11.8) |
| Sex, N (%) females  3DT1 Cervical CSA, mm ^2^, mean (SD) | | | | 70 (61.9%)  64.3 (8.99) |
| 3DT1 Brain measures, cm^3^, median (CI) | | | | |
|  | WMn | | | 449.8 (441.9, 465.1) |
|  | GMn | | | 650.1 (634.8, 662.5) |
|  | CGMn  DGMn | | | 610.9 (598, 623.1)  37.3 (36.5, 38.3) |

CSA: Cross sectional area; WMn: normalised white matter; GMn: normalised grey matter; CGMn: normalised cortical grey matter; DGMn: normalized deep grey matter.

**Supplementary Figure 1.** Flowchart of the included 3DFLAIR and 3DT1 Brain scans of the POINT-MS cohort


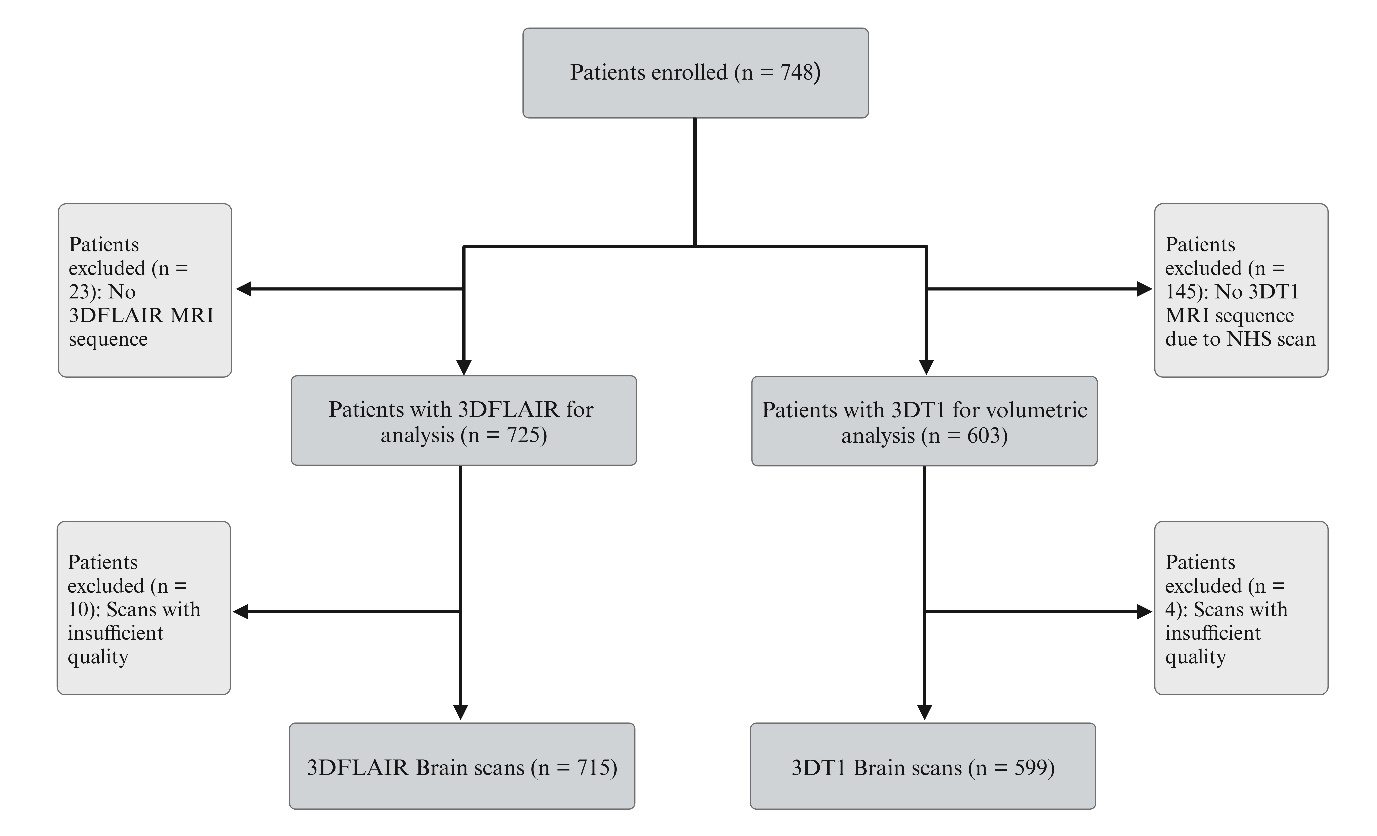


FLAIR: Fluid-Attenuated Inversion Recovery; MRI: Magnetic Resonance Imaging; NHS: National Health Service.
