# Supplementary material for: Single-centre, prospective cohort to predict optimal individualised treatment response in multiple sclerosis (POINT-MS): a cohort profile
Source: BMJ Open. 2025 Sep 25;15(9):e103440. doi: 10.1136/bmjopen-2025-103440 (PMC12481372; doi:10.1136/bmjopen-2025-103440)
Supplement: Supplementary file 2 [file bmjopen-2025-103440.reviewer_comments.pdf]

PEER REVIEW HISTORY

BMJ Open publishes all reviews undertaken for accepted manuscripts. Reviewers are asked to complete a checklist review form and are provided with free text boxes to elaborate on their assessment. These free text comments are reproduced below.

ARTICLE DETAILS

Title (Provisional)

Cohort profile: a single-centre, prospective cohort to predict optimal individualised treatment response in multiple sclerosis (POINT-MS)

Authors

Christensen, Ronja; Cruciani, Alessandro; Al-Araji, Sarmad; Bianchi, Alessia; Chard, Declan; Fourali, Samih; Hamed, Weaam; Hammam, Ahmed; He, Anna; Kanber, Baris; Maccarone, Davide; Moccia, Marcello; Mohamud, Suraya; Nistri, Riccardo; Passalis, Anestis; Pozzilli, Valeria; Prados Carrasco, Ferran; Samdanidou, Eirini; Song, Joy; Wingrove, Jed; Yam, Charmaine; Yiannakas, Marios; Thompson, Alan J; Toosy, Ahmed; Hacohen, Yael; Barkhof, Frederik; Ciccarelli, Olga; Aojula, Anuriti; Champsas, Dimitrios; Collorone, Sara; Eshaghi, Arman; Goodkin, Olivia; Kurd, Mohammad; Panella, Elena; Pansari, Fatima; Stutters, Jonathan; Toorop, Alyssa; Zhang, Le; Brownlee, Wallace; Chataway, Jeremy; Chung, Karen; De Angelis, Floriana; Leary, Siobhan; Khaleeli, Zhaleh; Wright, Sarah; Swanton, Josephine; Trip, Anand; Gandini Wheeler-kingshott, Claudia Angela; Solanky, Bhavana; Parker, Chloe; Stuart, Michaela; Haagenrud, Hanne; Wilson, Heather

---

VERSION 1 - REVIEW

Reviewer 1

Name Kearns, Patrick

Affiliation The University of Edinburgh Centre for Clinical Brain Sciences, Anne Rowling Regenerative Neurology Clinic

Date 10-Jun-2025

COI Non-financial association: I am an investigator in an unrelated similar cohort of patients with early MS recruited in Scotland.

This manuscript presents baseline data for the POINT-MS study: a large, prospective, single-centre observational cohort designed to address the prediction of individualised treatment response to disease-modifying therapies (DMTs). The authors provide a description of the study design, baseline assessment, and participant characteristics for an impressive 748 patients enrolled at Queen Square between 2019 and 2024. The study integrates clinical, neuropsychological, imaging (MRI and OCT), genetic, and serum biomarker data, with

longitudinal follow-up planned in five study visits for each participant occurring over five years. The rationale is clearly articulated, highlighting the variability in treatment response and the limitations of existing clinical predictors, with the overarching aim to enable precision medicine in MS care.

The baseline data are thoroughly described and the participant flow diagram in figure 1 is helpfully detailed and includes the number of subjects approached and reasons for non-inclusion in the final cohort. The data are adequately contextualised with respect to published trials of anti-CD20 therapies. Comparison to other observational real-world prospective cohorts (e.g. the long running EPIC and CLIMB studies, or the newer UK-based FutureMS study) or to registries (e.g. Danish MS registry, Italian MS registry, Norwegian MS registry, Swedish MS registry, Cemcat, Cleveland Clinic, British Columbia MS registry, MSBASE, MS Clinic Database Winnipeg etc.) is lacking. It would be helpful for the authors to frame their cohort in this context, as there are many disease registries that collect opportunistic data but relatively fewer true prospective observational cohort studies like POINT-MS. Comparisons with trial populations should be performed extremely cautiously as these populations are not intended to be representative.

Notable findings in the current manuscript include a high prevalence of cognitive impairment despite relatively low physical disability, a substantial proportion of patients initiating high-efficacy DMTs (particularly anti-CD20 therapies), and heterogeneity in MRI lesion load and spinal cord metrics. The high prevalence of ex- or current smokers at baseline reminds of the importance of addressing this potentially modifiable risk factor. The study’s strengths include its large n, deep clinical phenotyping, standardised imaging protocols, frequent biobanking and patient and public involvement in design. Embedding the study within NHS pathways promises to improve study retention and consent to access routine data offers opportunities to improve data completeness retrospectively. The manuscript effectively lays the foundation for future analyses, including predictive modelling, and offers a valuable resource for collaborative research into MS treatment stratification and disease progression mechanisms.

The study promises to be very valuable and the authors should be congratulated on the large number of recruited patients and the substantial amount of work that has gone into this. This cohort profile manuscript will form a reference document for the research team, and act as an advertisement to potential collaborators. For these reasons, I advise the authors consider the following comments:

Comments:

1. There is no methodological information about the genetic or biomarker experiments. The manuscript implies that serum and genetic biomarkers are measured at each follow up: "We collected demographic, lifestyle, socio-economic (i.e., income, occupational

levels and years of education), and clinical data, together with MRI, OCT, serum and

genetic biomarkers at baseline and each follow-up". This is potentially misleadingly worded. Presumably the authors mean that samples are biobanked at each study visit (as described in sample methods), but have not necessarily been analysed. It is usually not considered useful to measure genotype at multiple time points, but it depends on the form of genotyping which is unspecified. From the methods it appears that serum, plasma, and a single EDTA tube of whole blood are being banked at each time point? The language could be clarified to make it clear what future assays are planned even if there is some uncertainty to this.

2. Very limited information is presented about the planned statistical analyses or machine learning approaches. The study design poses some complexities here as it does not seem like any data will be collected for participants from before their enrolment in the study and so judging the impact of the intervention (DMT treatment) will be challenging without information on pre-treatment disease activity. Do the authors have a strategy to tackle this issue? Regression towards the mean will also be a concern given recruitment into the study is triggered by need for DMT initiation/escalation. Does the consent patient's give to access routinely collected data extend retrospectively to the period of time before recruitment in the study? Are the ethics explicit to allow the investigators to do this? This would be worth considering before the end of the study visits if not.

3. 41.8% of the cohort has had a DMT, are the authors collecting information on which DMTs have been previously used, durations, and timings. High vs low efficacy. Five individuals in the study have had five previous DMTs, therefore, it is assumed that some of these participants have had previous high efficacy therapies which would be expected to have some residual disease modifying effect after they have been switched. Same issues as above in point (2). Do the authors have a strategy for collecting important details about historical DMT usage.

4. One of the interesting findings of the paper is that a surprisingly high proportion of patients have cognitive impairment. Cognitive impairment is a feature of MS and the authors have performed an appropriate and wide battery of neuropsychological tests which are expected to be sensitive, so these results are potentially credible. However, the cognitive tests were converted to z-scores using a reference population and a standardised cut-off used to determine absolute judgement of impairment. How confident are the authors that this reference population is suitable? This should be explored as a systematic underscoring of z-scores is an alternative explanation.

5. It would be helpful if the authors would describe in more detail where they are in the follow up of patients (they expect to finish 5-year follow up in 2019, but how far through their five follow up visits are they for the cohort.) Study retention information and loss-to-follow up and reasons for LTF at each wave is important information, and how these data will be prospectively collected is important for downstream analyses. The authors should make some attempt to pre-specify how they will handle loss-to-follow up, efforts they will go to to

ensure study retention, how they will determine if a subject is lost to follow up at random, how they will ensure they identify subjects who have died and what they have died of, how they will handle loss to follow up in a subsequent wave (will efforts be made to re-recruit). Similarly, the authors should specify whether they are able to collect information on other healthcare interactions (e.g. admissions for serious infections), and whether they are collecting information on complications of therapies.

7. How will the authors handle diagnostic uncertainty that becomes apparent after recruitment. It would be helpful to detail the proportion of the cohort who have AQP4 and MOG antibodies tested at baseline if these data are available. Similarly the proportion with CSF analyses. Are these data collected/collectable in future?

8. The authors should consider how they will handle incidental findings. For example if the genetic study planned is SNP genotyping, then there are at least ten potentially actionable SNPs in seven genes BRCA1, BRCA2, ATP7B, TTN, KCNH2, MUTYH, GAA. There will also be anticipated incidental imaging findings. How will the authors handle these issues in patients that remain in the study. They should also consider how they will handle them in those who withdraw consent, move, or are otherwise lost to follow up.

9. Do the authors have data to help them understand what the cost of comparability is between the research MR scanner and the NHS scanner. Do they have a sense of what this will mean for comparison of results across study waves? I note that n=145 patients appear to have had NHS scans at baseline which do not have 3D T1 sequences. Have the authors previously (or intend to) systematically study the impact of scanner on key imaging outcomes. The inability to come for a study MRI may plausibly be impacted by disease activity or severity, so there is potential scope for confounding here that should be considered.

10. The description of the MR findings would benefit from inclusion of information about the distribution of intervals from the previous scans. E.g. "At baseline, 26% of participants

showed new T2 lesions in the brain and 8.5% in the spinal cord, compared to previous scans..."

11. Average disease duration is stated .as 95.2 months. How is this defined? Time from diagnosis or first symptom or composite?

**Reviewer**	**2**
**Name**	**Tozlu, Ceren**
**Affiliation**	**Weill Cornell Medicine**
**Date**	**24-Jun-2025**
**COI**	**None**

The POINT-MS cohort integrates longitudinal clinical, imaging, genetic, and serum biomarker data to improve the prediction of treatment response to disease-modifying therapies (DMTs) in people with MS. This large, prospective observational study is a valuable step toward advancing precision medicine in MS by identifying individual-level predictors of treatment efficacy and long-term disease outcomes. The use of multi-modal data and the substantial sample size are highly promising. Please find my comments and suggestions below:

• More clarification is needed on how cognitive impairment is identified. How are the cognitive tests z-scored, what is the description of the controls that were used to z-score (age, sex, education level distribution), and what was the threshold that was used to identify the patients with a cognitive impairment?

• It would be helpful to include the treatment duration, especially if there are patients who did not start DMT at the time of diagnosis.

• As MS impacts women more than men, therefore, women's health-related information is needed, such as the number of full-term pregnancies, the number of miscarriages, age at menopause onset, menopause symptoms, HRT use (dosage and duration), history of postpartum depression, etc. I recommend report these results if you collect, otherwise, recommend adding these questions to the follow-up questionnaires.

• Table 1 indicates White, Black, Asian, etc, as ethnicity; it should be corrected as race.

• "The racial and ethnic diversity of the cohort reflects the clinical catchment of central London." The ethnic information is not provided in the main text.

• It is unclear why CIS and RIS patients are excluded.

• Is the MRI collected with the same manufacturer and protocol? If not, a harmonization technique needs to be added to the main text.

• Detailed information about the MRI protocol is needed.

• What is the expected drop-off % based on these authors' experience with longitudinal studies?

• Do the authors aim to include other sequences, such as QSM, to classify the lesions as chronic active vs inactive, as PRLs were found to be associated with more aggressive disease progression, and it would be helpful to consider them for the DMT decisions.

• It is not clear how the lesion mask and individual lesions were created. Were they created manually, with LST, or a deep learning method? Did any author with lesion masking experience validate the final version of the lesion masks?

• Same question for the spinal cord lesions.

• It is not clear which kind of collaboration is encouraged for this cohort.

VERSION 1 - AUTHOR RESPONSE

Reviewer 1: Dr. Patrick Kearns, The University of Edinburg Centre for Clinical Brain Sciences, MRC Human Genetics Unit

This manuscript presents baseline data for the POINT-MS study: a large, prospective, single-centre observational cohort designed to address the prediction of individualised treatment response to disease-modifying therapies (DMTs). The authors provide a description of the study design, baseline assessment, and participant characteristics for an impressive 748 patients enrolled at Queen Square between 2019 and 2024. The study integrates clinical, neuropsychological, imaging (MRI and OCT), genetic, and serum biomarker data, with longitudinal follow-up planned in five study visits for each participant occurring over five years. The rationale is clearly articulated, highlighting the variability in treatment response and the limitations of existing clinical predictors, with the overarching aim to enable precision medicine in MS care.

The baseline data are thoroughly described and the participant flow diagram in figure 1 is helpfully detailed and includes the number of subjects approached and reasons for non-inclusion in the final cohort. The data are adequately contextualised with respect to published trials ofanti-CD20 therapies. Comparison to other observational real-world prospective cohorts (e.g. the long running EPIC and CLIMB studies, or the newer UK-based FutureMS study) or to registries(e.g. Danish MS registry, Italian MS registry, Norwegian MS registry, Swedish MS registry, Cemcat, Cleveland Clinic, British Columbia MS registry, MSBASE, MS Clinic Database Winnipeg etc.) is lacking. It would be helpful for the authors to frame their cohort in this context, as there are many disease registries that collect opportunistic data but relatively fewer true prospective observational cohort studies like POINT-MS.

Comparisons with trial populations should be performed extremely cautiously as these populations are not intended to be representative.

Notable findings in the current manuscript include a high prevalence of cognitive impairment despite relatively low physical disability, a substantial proportion of patients initiating high-efficacy DMTs (particularly anti-CD20 therapies), and heterogeneity in MRI lesion load and spinal cord metrics. The high prevalence of ex- or current smokers at baseline reminds of the importance of addressing this potentially modifiable risk factor. The study's strengths include its large n, deep clinical phenotyping, standardised imaging protocols, frequent biobanking and patient and public involvement in design. Embedding the study within NHS pathways promises to improve study retention and consent to access routine data offers opportunities to improve data completeness retrospectively. The manuscript effectively lays the foundation for future analyses, including predictive modelling, and offers a valuable resource for collaborative research into MS treatment stratification and disease progression mechanisms.

Response: Thank you for your positive comments and constructive feedback. We agree that situating the POINT-MS cohort within the broader landscape of registries and prospective cohorts strengthens the context and underlines the novelty of our cohort. We have now revised the discussion section to include a brief comparative overview as follows (we have kept the anti-CD20 trials as these included patients initiating a DMT):

p. 72, l. 47.

While many MS data cohorts exist globally, including large scale (inter)national registries (e.g., MSBASE, the Danish, Italian and Swedish MS Registries), these are typically retrospective and often rely on opportunistic and variable clinical data. In contrast, POINT-MS includes a deeply phenotyped, prospective cohort with harmonised clinical, imaging and blood/serum collection protocols across follow-up. This design aligns more closely with other prospective single-centre research cohorts, such as the EPIC, CLIMB, and FutureMS cohorts. Compared to these, POINT-MS is unique in its systematic multi-modal integration, real-world treatment setting, and prevalence of high-efficacy DMT initiators. Importantly, POINT-MS provides complementary data to clinical trial populations, particularly those in antiCD20 studies, while still reflecting real-world diversity and treatment patterns.

p. 69, l. 8.

The baseline characteristics of the POINT-MS cohort revealed interesting observations and align closely with those of some single-centre or multi-centre (national) cohorts and anti-CD20 trials in RRMS. In particular, patients' age (mean 48.8 yrs (SD 10.9) in our cohort) was similar to that of the EPIC (UCSF, USA) cohort (mean 41.6 yrs (SD 9.7)[35], FutureMS (Scotland) cohort (mean age at diagnosis 37.7 yrs) 36, and anti-CD20 trials (37.1 yrs in the ocrelizumab trial[4] and 38.9 yrs in the ofatumumab trial[37]).The percentage of female patients was also similar (68% in our cohort, 70.4% in the EPIC cohort, 75.4% in the FutureMS cohort).[4,37]. POINT-MS patients' disability was mild (median EDSS=2), which was the same as that of patients in the FutureMS cohort, and similar to that of patients enrolled in the EPIC cohort (median EDSS 1.5), ocrelizumab (mean 2.86) and ofatumumab (mean 2.97) trial, although the EDSS range in the POINT-MS cohort was wider (up to EDSS 8).

..

The study promises to be very valuable, and the authors should be congratulated on the large number of recruited patients and the substantial amount of work that has gone into this. This cohort profile manuscript will form a reference document for the research team, and act as an advertisement to potential collaborators. For these reasons, I advise the authors consider the following comments:

Comment 1: There is no methodological information about the genetic or biomarker experiments. The manuscript implies that serum and genetic biomarkers are measured at each follow up: "We collected demographic, lifestyle, socio-economic (i.e., income, occupational levels and years of education), and clinical data, together with MRI, OCT, serum and genetic biomarkers at baseline and each follow-up". This is potentially misleadingly worded. Presumably the authors mean that samples are biobanked at each study visit (as described in sample methods), but have not necessarily been analysed. It is usually not considered useful to measure genotype at multiple time points, but it depends on the form of genotyping which is unspecified. From the methods it appears that serum, plasma, and a single EDTA tube of whole blood are being banked at each time point? The language could be clarified to make it clear what future assays are planned even if there is some uncertainty to this.

Response 1: We thank the reviewer for this insightful comment. We agree that our original wording could have been misleading and have revised the manuscript to clarify our sample collection. Specifically, we now clearly state that our plan is to carry out both genetic analysis (at baseline) and epigenetic analysis (over time).

p. 55, l. 15.

"Blood and serum samples were bio-banked at each visit, enabling future biomarkers and both genetic (i.e., baseline) and epigenetic analyses."

Comment 2: Very limited information is presented about the planned statistical analyses or machine learning approaches. The study design poses some complexities here as it does not seem like any data will be collected for participants from before their enrolment in the study and so judging the impact of the intervention (DMT treatment) will be challenging without information on pre-treatment disease activity. Do the authors have a strategy to tackle this issue? Regression towards the mean will also be a concern given recruitment into the study is triggered by need for DMT initiation/escalation. Does the consent patient's give to access routinely collected data extend retrospectively to the period of time before recruitment in the study? Are the ethics explicit to allow the investigators to do this? This would be worth considering before the end of the study visits if not. **Comment 3:** 41.8% of the cohort has had a DMT, are the authors collecting information on which DMTs have been previously used, durations, and timings. High vs low efficacy. Five individuals in the study have had five previous DMTs, therefore, it is assumed that some of these participants have had previous high efficacy therapies which would be expected to have some residual disease modifying effect after they have been switched. Same issues as above in point (2). Do the authors have a strategy for collecting important details about historical DMT usage?

Response to Comments 2 and 3: We thank the reviewer for highlighting the importance of accounting for historical DMT use, including timing, duration, and efficacy classification. We agree that these are key variables that influence disease trajectory and treatment response. Our strategy was to collect at baseline detailed retrospective treatment history for patients who were not treatment naive but were switching from a different DMT. In these patients we collected information on start/stop dates for each DMT, DMT type/dosage, and reason for treatment discontinuation. Using this data, we have updated the manuscript to include proportion of disease duration on DMT (any DMT, high efficacy and non-high efficacy), which is now reported in the new Table 1 and in the results section, as below. We also confirm that ethical approval (23/WS/0008) permits retrospective access to clinical records, enabling us to collect relevant pre-enrolment data, including DMT exposure from diagnosis onward. These data will be incorporated into planned longitudinal analyses and predictive models to account for prior treatment history and potential carryover effects.

p. 66, l. 10.

At study entry, 59.2% of participants were treatment-naïve, and 83.2% initiated a high-efficacy DMT. The drug which was most frequently initiated was ocrelizumab (51.4%), followed by ofatumumab (30.1%) (**Figure 3**). At baseline, participants had spent an average of 22.7% (SD 31.6%) of their disease duration (calculated from symptom onset until day of baseline visit) on any DMT. The average proportion of time spent on high-efficacy DMTs was 3.9% (SD 12.1%), while 18.8% (SD 30.3%) of disease duration was spent on non-high efficacy DMTs (**Table 1**).

Comment 4: One of the interesting findings of the paper is that a surprisingly high proportion of patients have cognitive impairment. Cognitive impairment is a feature of MS and the authors have performed an appropriate and wide battery of neuropsychological tests which are expected to be sensitive, so these results are potentially credible. However, the cognitive tests were converted to z-scores using a reference population and a standardised cut-off used to determine absolute judgement of impairment. How confident are the authors that this reference population is suitable? This should be explored as a systematic underscoring of z-scores is an alternative explanation.

Response 4: We agree and in fact have been collecting neuropsychological data in our own healthy control cohort. This also aligns with BICAMS official guidelines (Langdon et al., 2012), that recommend using country-specific normative data.

We have now repeated the calculation of z-score using our own control data. Interestingly, when compared to the results obtained with the reference population, z-scoring with our own control data (N = 53) changed our results showing a lower impairment rate on SDMT (28% with our own control group vs. 52.1% when using the reference population) and BVMT-R (24% with our own group vs. 32.9% when using the reference population), and a higher impairment on CVLT-II (17.6% vs. 5.3%).

We have made changes accordingly to methods, results and discussion (and updates to Table 1, Figure 2, abstract, and added another supplementary material not pasted here).

Methods p. 61, l. 41.

Healthy controls were recruited as part of a larger longitudinal study on cognitive function in MS, approved by the UCL Research Ethics Committee (Ethics number: 28405/002), and written consent was obtained from all participants. Age, sex, and years of education were used to generate demographically adjusted z-scores for each patient based on our own healthy control sample (N = 53; mean age 30 yrs (SD 13), years of education 15.21 (SD 2.44) and 57% female (see Supplementary Materials for procedure).~~on available normative data~~ [24]

Results p. 65, l. 47.

On BICAMS test of cognitive performance, 301 (40.2%)~~62~~ of the participants showed impairment in at least one test (**Table 1**); 28~~51.1~~% were classified as impaired on the SDMT, 24~~32.3~~% on the BVMT-R, while~~ only~~ 17.6~~5.2~~% scored below the cut-off on the CVLT-II. When comparing impairments between the BICAMS subtests, 10~~27~~.7~~3~~% of participants were impaired only on the SDMT, 7.1~~8.6~~% on BVMT-R, and 4.1~~0.7~~% (N = 31~~5~~) on CVLT-II, while 5.8~~20.7~~% were impaired on both SDMT and BVMT-R, 2.5~~1~~% (N = 19~~7~~) on both SDMT and CVLT-II, while 2.7~~0.6~~% (N = 20~~4~~) impaired on both BVMT-R and CVLT-II **(Figure 2).**

Discussion p. 69, l. 43.

The POINT-MS cohort showed a ~~high~~ 40% rate of cognitive impairment, defined as failure on a single test. This~~, which~~ agrees with previous reports [36,37] ~~.~~ and underscore the substantial cognitive burden even among patients with relatively mild physical disability (as assessed by the EDSS).

Comment 5: It would be helpful if the authors would describe in more detail where they are in the follow-up of patients (they expect to finish 5-year follow up in 2019, but how far through their five follow up visits are they for the cohort.) Study retention information and loss-to-follow up and reasons for LTF at each wave is important information, and how these data will be prospectively collected is important for downstream analyses. The authors should make some attempt to pre-specify how they will handle loss-to-follow up, efforts they will go to to ensure study retention, how they will determine if a subject is lost to follow up at random, how they will ensure they identify subjects who have died and what they have died of, how they will handle loss to follow-up in a subsequent wave (will efforts be made to re-recruit). Similarly, the authors should specify whether they are able to collect information on other healthcare interactions (e.g. admissions for serious infections), and whether they are collecting information on complications of therapies.

Response 5: We appreciate the reviewer's comment regarding the importance of detailed information on patient follow-up, retention, and loss to follow-up (LTF). While this manuscript does not focus on these longitudinal aspects in depth, we have added the following paragraph to the Results section:

June 2025 update on the POINT-MS cohort

Out of 748 patients seen at baseline, 704 (94%) have been seen at 6–12 months, 544 (73%) at 18–24 months, and 84 (11.2%) at 54–60 months (the latter two time points are still ongoing). Reasons for missed visits are being recorded and reviewed by the study team. As previously noted, ethical approval (REC 23/WS/0008) allows retrospective access to electronic health records, enabling us to fill in data gaps (e.g., EDSS scores from clinical appointments, NHS-acquired MRIs) and extract additional clinical outcomes (e.g., serious infections, hospital admissions, or cause of death) for participants who remain under the care of our NHS Trust but do not attend study visits. For participants truly lost to follow-up—such as those who move abroad or transfer to a different NHS Trust—appropriate methods for handling missing data will be selected based on the study's analytical objectives, and sensitivity analyses will be conducted accordingly.

Comment 6: How will the authors handle diagnostic uncertainty that becomes apparent after recruitment. It would be helpful to detail the proportion of the cohort who have AQP4 and MOG antibodies tested at baseline if these data are available. Similarly the proportion with CSF analyses. Are these data collected/collectable in future?

Response 6: Participants are enrolled after a diagnosis of MS is confirmed by their treating neurologist, typically following standard diagnostic investigations conducted at UCLH, including MRI, CSF analysis and antibody testing where indicated. AQP4 and MOG antibody testing is part of routine diagnostic work-up in relevant clinical scenarios, and CSF analysis is performed in the majority of cases, especially for excluding alternative diagnoses and when applying the McDonald diagnostic criteria. We have not calculated the percentage of patients in the POINT-MS cohort who underwent a CSF analysis, but these data are potentially available on our electronic health records. In the unlikely event that a patient is later diagnosed with an alternative condition, they will be excluded from the cohort (and this cause will be recorded). To this date, no patients have been excluded for this reason.

Comment 7: The authors should consider how they will handle incidental findings. For example if the genetic study planned is SNP genotyping, then there are at least ten potentially actionable SNPs in seven genes BRCA1, BRCA2, ATP7B, TTN, KCNH2, MUTYH, GAA. There will also be anticipated incidental imaging findings. How will the authors handle these issues in patients that remain in the study. They should also consider how they will handle them in those who withdraw consent, move, or are otherwise lost to follow up.

Response 7: Thank you for raising this important ethical consideration. Our approach to incidental findings follows the Ethical approvals for this study.

For genetic data, participants are explicitly informed during the consent process that analysis is conducted solely for research purposes and that no individual results will be returned. This is in line with guidance from UCL's research ethics committees and mirrors the approach taken by other large-scale research initiatives, such as the UK Biobank. For MRI data, incidental findings are handled on a case-by-case basis. Structural MRI scans are reviewed to

exclude participants with significant comorbid neurological conditions or unexpected pathology that could interfere with study outcomes. Clinically significant findings, particularly those not previously known to the participant, are assessed by the study team and, where necessary, flagged for clinical review according to UCLH policy. Incidental findings in patients who are lost at follow-up are also recorded.

Comment 8: Do the authors have data to help them understand what the cost of comparability is between the research MR scanner and the NHS scanner. Do they have a sense of what this will mean for comparison of results across study waves? I note that n=145 patients appear to have had NHS scans at baseline which do not have 3D T1 sequences. Have the authors previously (or intend to) systematically study the impact of scanner on key imaging outcomes. The inability to come for a study MRI may plausibly be impacted by disease activity or severity, so there is potential scope for confounding here that should be considered.

Response 8: Thank you for raising this. We acknowledge the challenges associated with scanner variability and the absence of 3D T1 sequences in a subset of NHS baseline scans (n = 145) and at follow-up visits. Importantly, the 3D FLAIR sequence is implemented across sites, as part of the MS standard-of-care MRI protocol. We are currently investigating the use of the 3D FLAIR sequence – which is implemented across all sites as part of the NHS MS-care protocol and included in our research protocol –for estimating brain volume and lesion burden. This approach will be informed by previous work using GIF-based methods (e.g., Goodkin et al., 2021, https://doi.org/10.1016/j.nicl.2020.102542).

We also acknowledge that the inability to come for a study MRI may be influenced by the disease severity or mobility limitations. This introduces a potential source of bias, which we plan to explore in future analyses by comparing clinical characteristics of participants who did and did not attend research MRIs; this analysis will be possible because we will be able to find the required information in the electronic health records.

Comment 9: The description of the MR findings would benefit from inclusion of information about the distribution of intervals from the previous scans. E.g. "At baseline, 26% of participants showed new T2 lesions in the brain and 8.5% in the spinal cord, compared to previous scans..."

Response 9: We agree and have now updated the results section to include mean intervals separately for patients with and without new brain or spinal cord lesions, as follows:

p. 67, l. 15.

“Previous imaging was available for comparison in 668 patients (89.3%). At baseline, 26% of patients had new brain lesions and 8.5% had new spinal cord lesions compared to their most recent clinical scans. The mean interval since the prior scan was 9 months (SD 8.2; range 0–80 months). Among patients with new brain lesions, the mean interval was 10.4 months (SD 9.4), compared to 9.0 months (SD 7.4) in those without new lesions. For new spinal cord lesions, the corresponding mean intervals were 10.8 months (SD 9.9) in patients with new lesions versus 9.0 months (SD 8.0) in those without.

<u>Comment 10:</u> Average disease duration is stated .as 95.2 months. How is this defined? Time from diagnosis or first symptom or composite?

<u>Response 10:</u> Apologies for the lack of clarity – disease duration is defined as time since symptom onset. We have corrected this in text as follows and add a note to Table 1.

p. 53, l. 52.

"They were mostly female (68%) and White (75%) participants, with relapsing-remitting MS (94.3%), and with an average age of 40.8 (±10.9) years and a mean disease duration of 7.9 (±7.4) years **since symptom onset**."

p. 65, l. 33

"Patients had a relatively short disease duration (mean 7.9, SD: 7.4 years **since symptom onset**)."

Reviewer 2: Dr. Ceren Tozlu, Weill Cornell Medicine

The POINT-MS cohort integrates longitudinal clinical, imaging, genetic, and serum biomarker data to improve the prediction of treatment response to disease-modifying therapies (DMTs) in people with MS. This large, prospective observational study is a valuable step toward advancing precision medicine in MS by identifying individual-level predictors of treatment efficacy and long-term disease outcomes. The use of multi-modal data and the substantial sample size are highly promising. Please find my comments and suggestions below:

<u>Comment 1</u>: More clarification is needed on how cognitive impairment is identified. How are the cognitive tests z-scored, what is the description of the controls that were used to z-score (age, sex, education level distribution), and what was the threshold that was used to identify the patients with a cognitive impairment?

<u>Response 1:</u> We thank the reviewer for raising this point, which was also raised by reviewer 1. Please note that we have now changed our reference population, which notably changed impairment rates from those originally calculated from published normative data (Parmenter et al., 2010). Please see response to Reviewer 1, Comment 4 for the full details of the changes. In summary, when compared to the results obtained with the reference population, z-scoring with our own control data (N = 53) changed our results showing a lower impairment rates on SDMT (28% with our own control group vs. 52.1% when using the reference population) and BVMT-R (24% with our own group vs. 32.9% when using the reference population), and a higher impairment on i CVLT-II (17.6% vs. 5.3%).

Please also see changes in the Methods (p. 61, l. 41), Results (p. 65, l. 47), and Discussion (p. 69, l. 42).

p. 61, l. 55

A test score was classified as impaired if it fell below ≥1.5 standard deviations from the mean

..

Further information on the threshold is given in the Supplementary Material S1 (page 92), and it is as follows:

Cognitive impairment was defined as a z-score less than -1.5 on any given test (i.e., ≥1.5 SD below the demographically adjusted mean. This threshold is commonly used in neuropsychological research and reflects clinically meaningful deviation from expected cognitive performance. Group-level impairment was further categorised as “Impaired” or “Preserved” for each subtest based on this cut-off.

Comment 2: It would be helpful to include the treatment duration, especially if there are patients who did not start DMT at the time of diagnosis.

Response 2: Thank you for raising this suggestion. As noted in our response to Reviewer 1 (Comments 2 and 3), we have collected detailed retrospective DMT histories for all participants, including start and stop dates for each treatment, allowing us to calculate the duration of time spent on DMTs relative to disease duration. These values (i.e., proportion of disease duration from symptom onset to baseline date on 1) any DMT, 2) high-efficacy, and 3) non-high efficacy DMTs) are now reported in the new Table 1 and described in the Results section (see p. 66, l. 15).

Comment 3: As MS impacts women more than men, therefore, women’s health-related information is needed, such as the number of full-term pregnancies, the number of miscarriages, age at menopause onset, menopause symptoms, HRT use (dosage and duration), history of postpartum depression, etc. I recommend report these results if you collect, otherwise, recommend adding these questions to the follow-up questionnaires.

Response 3: We thank the reviewer for this thoughtful and important comment. We agree that reproductive and hormonal health information is highly relevant given the disproportionate impact of MS on women. We confirm that this information was collected cross-sectionally for female participants, including number of full-term pregnancies, number of miscarriages, age at menarche/menopause, etc. We have revised the manuscript to clearly indicate that female patients were invited to provide this information, and included high-level descriptive statistics in the new Table 1 (further granularity and additional analyses of these data will be provided in future publications), as follows:

p. 64, l. 8.

In addition, all female participants were asked targeted women’s health questions cross-sectionally, including age of menarche/menopause, pregnancy history, miscarriage history, HRT and contraceptive use.

Comment 4: Table 1 indicates White, Black, Asian, etc, as ethnicity; it should be corrected as race.

Response 4: Thank you for this comment. In the UK (e.g., NHS, Office for National Statistics) Asian, Black, White, and Mixed are classified under ethnic groups, not "race." These classifications are used in censuses and other official data collections, providing a framework for understanding the ethnic composition of the UK population. We are aware that the U.S. Census separates race and ethnicity, with "Hispanic or Latino" treated as ethnicity, and White, Black or African American, Asian, American Indian/Alaska Native, etc. as racial groups. Therefore, since this is a UK-based study, we had used the term ethnic group. Since this could be confusing to the non-UK readers, we have added Racial to Ethnic groups.

Comment 5: "The racial and ethnic diversity of the cohort reflects the clinical catchment of central London. "The ethnic information is not provided in the main text.

Response 5: We have amended as follows:

p. 70, l. 42.

"The racial/ethnic ~~and ethnic~~ diversity of the cohort reflects the clinical catchment of central London. Of the participants, 189 (24.9%) identified themselves as belonging to a non-White ethnic group, with Asian and Black individuals being the most represented among them".

Comment 6: It is unclear why CIS and RIS patients are excluded.

Response 6: This is because we included patients who initiated a new DMT within 3 months (see inclusion criteria *(page 60, l. 24… (2) Initiation of a new DMT within 3 months…),* and therefore we were unable to include CIS/RIS patients as they are not eligible for DMT initiation in the NHS in the UK.

Comment 7: Is the MRI collected with the same manufacturer and protocol? If not, a harmonization technique needs to be added to the main text.

Response 7: Thank you for this comment. For this cross-sectional baseline study, we analysed only 3D T1-weighted research scans (N=599), which was acquired with the same sequences and the same 3T Philips scanner, and thus, a harmonisation technique was not needed. However, this is a very important point for future longitudinal analyses, where we plan to apply the ComBat method (Orlhac et al., 2022, DOI: 10.2967/jnumed.121.262464) to harmonise MRI measurements across sites. This approach requires no additional scans and corrects for site effects by aligning the means and standard deviations of parameter distributions. A Kolmogorov-Smirnov test will be used to assess initial site differences, the need for correction, and the effectiveness of harmonisation.

We have added the following comment to the results:

p. 67, l. 35.

We obtained volumetric brain measures using only the 3D-T1 research scans (N=599, since in 4 subjects the analysis failed because of movement artifacts); none of the NHS scans include 3D-T1.

Comment 8: Detailed information about the MRI protocol is needed.

Response 8: We have provided the most relevant MRI parameters for the MRI protocol acquired on a 3T Philips scanner (N=599) in Supplementary Table 2.

Comment 9: What is the expected drop-off % based on these authors' experience with longitudinal studies?

Response 9: Based on our experience with this and similar longitudinal studies, we anticipate a cumulative drop-out rate of 5-10% a year. This estimate aligns closely with our observed retention so far: Out of 748 patients seen at baseline, 704 (94%) have been seen at 6–12 months, 544 (73%) at 18–24 months, and 84 (11.2%) at 54–60 months (the latter two time points are still ongoing). Reasons for missed visits are being recorded and reviewed by the study team.

While the absolute numbers continue to evolve, we are actively tracking reasons for attrition and expect that the final five-year retention will remain within this projected range. Additionally, we plan to use retrospective data linkage (as permitted by ethical approval 23/WS/0008) to capture missing outcomes which are routinely done in clinic. These methods will help mitigate bias due to cases lost to follow-up and support robustness of longitudinal analyses.

Comment 10: Do the authors aim to include other sequences, such as QSM, to classify the lesions as chronic active vs inactive, as PRLs were found to be associated with more aggressive disease progression, and it would be helpful to consider them for the DMT decisions.

Response 10: Thank you for this comment. While we agree that other sequences would provide specificity and granularity for our estimation of treatment response, this was beyond the scope of this project, and currently, we do not plan to incorporate further MRI sequences in the protocol.

Comment 11: It is not clear how the lesion mask and individual lesions were created. Were they created manually, with LST, or a deep learning method? Did any author with lesion masking experience validate the final version of the lesion masks?

Response 11: We thank the reviewer for this important question. Lesion masks were generated using the automated lesion delineation software nicMS (Valverde et al., 2017 doi:10.1016/j.neuroimage.2017.04.034), which uses a supervised learning algorithm to identify white matter lesions from 3D T2-FLAIR images. All resulting lesion masks were

manually reviewed and adjusted as needed by trained analysts with experience in lesion masking, under the supervision of a senior neuroradiologist (F.B). This quality control step ensured the accuracy and reliability of the final lesion masks used for lesion volume quantification. We have amended the manuscript, as follows:

p. 62, l. 36.

The images were pre-processed as follows: (i) Lesions were identified using the automated lesion delineation software, nicMS [25] , with three-dimensional (3D) T2-Fluid Attenuated Inversion Recovery (FLAIR) images as input; all lesion masks were manually reviewed and adjusted as needed by trained analysts under the supervision of a senior neuroradiologist (FB), and lesion load was calculated as the total volume of focal lesions. (ii)

Comment 12: Same question for the spinal cord lesions.

Response 12: Similarly, we have amended the spinal segmentation section as follows:

p. 63, l. 13.

(v) For the computation of upper cervical spinal cord CSA 3D-T1 images of the brain were used [27 ;] in brief, the top of C2 vertebral body and the bottom of C3 vertebral body were first manually marked by trained analysts and the CSA was subsequently segmented automatically using the DeepSeg algorithm available with the Spinal Cord Toolbox (SCT) [27] .

Comment 13: It is not clear which kind of collaboration is encouraged for this cohort.

Response 13: We have now clarified the types of collaboration that we aim to encourage. We particularly welcome collaborations that support data sharing, pooled analysis or contributions that help extend the longitudinal follow-up or external validation of emerging predictive tools. Our goal is to maximise the scientific value and translational impact of the POINT-MS dataset.

p. 73, l. 34.

We welcome potential collaborators to engage with the POINT-MS study or related research on individualised treatment response and mechanisms of disability in MS. This includes opportunities for data sharing, pooled or harmonised analyses, and hypothesis-driven sub-studies, as well as collaborative contributions to imaging, clinical, and biomarker research. ~~We are also keen to expand on these aspects with a larger cumulative sample size~~. Anonymised and tabulated data from the POINT-MS cohort can be requested by contacting the principal investigator (OC).

VERSION 2 - REVIEW

**Reviewer**	**1**
**Name**	**Kearns, Patrick**

Affiliation **The University of Edinburgh Centre for Clinical Brain Sciences, Anne Rowling Regenerative Neurology Clinic**

Date **20-Aug-2025**

COI

The authors have provided detailed and considered responses to all comments. No further comments.

Reviewer **2**

Name **Tozlu, Ceren**

Affiliation **Weill Cornell Medicine**

Date **17-Aug-2025**

COI

I thank the authors for their response and for editing the manuscript based on the editors' and reviewers' comments. I don't have further comments. I suggest accepting it as is.
